# Spatial Color Efficacy in Perceived Luxury and Preference to Stay: An Eye-Tracking Study of Retail Interior Environment

**DOI:** 10.3389/fpsyg.2020.00296

**Published:** 2020-03-31

**Authors:** Ji Young Cho, Joori Suh

**Affiliations:** ^1^Department of Housing and Interior Design, Kyung Hee University, Seoul, South Korea; ^2^School of Architecture and Interior Design, University of Cincinnati, Cincinnati, OH, United States

**Keywords:** color, interior design, luxury, preference, retail

## Abstract

Color is a significant interior element with the power to influence emotions and behaviors in a particular environment. Numerous studies have investigated the impact of a single color on emotion; however, the collective emotional, cognitive, and behavioral effect created by combinations of colors applied to a space has not been thoroughly investigated. In this study involving both a survey as well as eye-tracking technology, we explored shaping the concept of spatial color efficacy by examining different applications of the same color combination in a space to determine whether they may cause different emotional responses, thereby impacting viewers’ perception of luxury and intention to stay. A total of 26 interior design students at a university in Korea participated in the study. An environment simulating a hypothetical retail store was developed using a 3D rendering program, and six variations of spatial applications were created for each high luxury color combination and low luxury color combination to be used as stimuli. While viewing the images, participants were asked to identify which image looked most luxurious and in which space would they most want to stay. Results show the following: (a) the same color combination, if applied differently in a physical environment, can create different emotional responses, thereby affecting perceived luxury and preference to stay; (b) even a low luxury color combination can enhance perceived luxury and preference to stay depending on the spatial application; (c) gaze bias exists when selecting the most luxurious space and stating preference to stay as shown in the high correlation between dwell time and choice; in addition, differences in emotional response across images were also observed in the variations of pupil sizes measured during viewing various applications; (d) dark colors used in large amounts of surface were perceived as more luxurious than light colors when the same color combination was applied; and (e) appropriate contrast among colors was more influential in preference to stay than extreme or minimal contrast. Results expand the understanding of human behavior in relation to spatial color efficacy based on the spatial color combination and potential decision-making process in a retail setting.

## Introduction

Color is a significant interior element with the power to influence emotions, such as pleasure and arousal; cognition, such as meaning making; and behaviors, such as intention to approach or avoid, in a particular environment (Spence et al., 2014). A retail store environment is a significant marketing point to deliver a brand’s intended image and message (Cho and Lee, 2017), and the perception of luxury is known to yield pleasure and positive emotion (Yani-de-Soriano and Foxall, 2006). Although numerous studies on the impact of color on emotion and behavior have been conducted, most studies have involved a single color, and few researchers have attended to the effect of the combination of several colors (Deng et al., 2010). Moreover, the question of human emotional, cognitive, and behavioral responses to variations of spatial application, such as on walls, floors, or furniture, has not been clearly answered. If applied differently in a space, will a color combination yield different emotional responses? What spatial application of color results in a high level of perceived luxury (PL) or preference to stay (PS)? The term *efficacy* refers to “the power to produce a desired result or effect” (Efficacy, 2020). In this research, we define the term *spatial color efficacy* as “the power of the ambiance created by a three-dimensional space, where a certain color combination is applied, to produce a desired emotional, cognitive, and behavioral effect.” Spatial color efficacy could range from highly effective to not at all effective.

The contemporary culture of emphasizing individual identity has resulted in the tendency to prefer higher-quality products that express distinctiveness, elitism, and uniqueness. The concept of luxury has been studied in a wide range of disciplines, including marketing, branding, consumer science, and design. Although the meaning of luxury may vary per individual and culture because it is multidimensional, literature shows that PL consists of the following five dimensions: conspicuousness, uniqueness, quality, hedonism, and extended self (Vigneron and Johnson, 2004). Conspicuousness is associated with wealth and elitism; uniqueness refers to exclusiveness and rarity; quality means well-crafted and superior; hedonism refers to glamorous and stunning; and extended self means being successful and powerful (Vigneron and Johnson, 2004, p. 487). In hedonic dimensions, Dubois and Laurent (1994) stated that the key elements of luxury are the pleasure, satisfaction, and excitement experienced when purchasing luxurious products.

Recently, the concept of luxury has migrated from the traditional understanding of exclusivity, the privilege of royalty, and inaccessibility to the contemporary notion of an accessible medium for self-expression, reflecting a “democratic right to happiness” (Chandon et al., 2016, p. 299). In view of the growth of the culture of contemporary millennials and their penchant for multidimensional experiences, opportunities for self-exploration, and novelty, a deeper understanding of luxury experience in terms of emotional stimulation and sensorial discoveries becomes crucial (de Kerviler and Rodriguez, 2019). When a luxury brand provides experience with emotional, sensorial, and intellectual stimulation, such experience broadens one’s self-concept and sense of self (de Kerviler and Rodriguez, 2019). The implication is that the concept of luxury has been expanded to customers’ long-term emotional well-being. Previous studies have shown that the feeling of luxury can draw positive emotion, such as pleasure and arousal; therefore, designing a retail space that expresses luxury would benefit retailers in that creating such a space for customers would make them feel inclined to stay (Cho and Lee, 2017).

In fact, while online shopping continues to grow, customers who patronize brick-and-mortar stores still look for meaningful emotional experiences that cannot be provided online (Kim and Lee, 2016). Kotler (1973) argued in his seminal article “Atmospherics as a Marketing Tool” that sensory qualities of the retail space influence buyers’ perception of the atmospheric quality of the space, and the affective state of buyers may eventually impact purchase probability. Offline stores are no longer merely spaces for selling products; instead offline retailers have discovered the need for strategies to provide sensuous and experiential spaces where customers desire to stay longer. The physical environment of a store affects the emotional experience of consumers and in turn influences their behavior and attitude (Donovan and Rossiter, 1982; Middlestadt, 1990; Kim and Lee, 2016). Important in causing emotional, cognitive, and behavioral responses, a physical environment where a service takes place is called a servicescape, which is “a composite of three dimensions: ambient conditions; spatial layout and functionality; and signs, symbols, and artifacts” (Bitner, 1992, p. 65). With the surge in the development of interactive art, highly visual digital culture, and high-end technology, designers have become responsible for thoughtful visual control in their proposal of emotional and sensorial stimuli in spatial design.

When entering a space, people make emotional and cognitive evaluations based on the information they receive from the physical spatial environment (Rye et al., 2016). Whether the emotional or cognitive state occurs first is debatable and unresolved, with many researchers arguing the two perspectives (Lin, 2004). In an extensive review of the cognition–emotion debate, Lin (2004) concluded that in evaluating a physical environment, such as a servicescape, cognitive processing in organizing the perceptual image of the environment occurs before affective processing, followed by the other cognitive processing, which is a more concrete evaluation of the environment. Finally, behavioral response, such as approach or avoidance, proceeds. This implies that sensory stimuli in a retail store are first perceived by a customer and affect her or his emotional state of pleasure–arousal. The emotional state influences the customer’s cognitive evaluation of the environment, that is, their perception of friendliness, luxury, or PS, which can lead to the customer’s behavioral responses of approach or avoidance and make a purchase. Typically, an interior physical environment is understood as structural interior components, such as ceiling, floor, walls, columns, or stairs; and non-structural spatial elements, such as color, materials, light fixtures, and decorative items that shape the spatial image (Cho and Suh, 2020). One of the main sensory channels used by humans is sight; thus, color, size, and shape are the main visual dimensions of an atmosphere (Kotler, 1973). Among these, color is the most expressive in its properties, and the immediate nature of its delivery of information accounts for the perception of color playing an important role in retail stores, shaping the image of the overall interior space, impression, and meaning (Söker, 2009; Kim and Kim, 2017). Color creates psychological as well as physiological effects, causing various emotional feelings as well as value judgments and intention to buy (Baker et al., 2002; Ou et al., 2004). Many researchers who have focused on the atmospheric aspects of color in relation to the enhancement of the shopping experience have tried to draw broad conclusions about the effect of a single color. To illustrate, the majority of studies on a single hue have dealt with the impact of colors with contrasting color temperature (e.g., cool vs. warm) or those with different wavelengths (e.g., long vs. short) on arousal or pleasure; but because the interior space where people reside is not typically monochromatic, understanding the impact of color combination on emotion requires additional attention.

Studies using color combination are limited and still in their early stages. *Color Image Scale* by Japanese psychologist, Kobayashi (1990), includes an extensive set of three-color combinations associated with certain adjectives, such as calm or vivid. Kobayashi provided 1,170 three-color combinations associated with 180 adjectives based on surveys and questionnaire, but the combinations are provided in palettes and the detailed research procedures and findings were not reported. The research of Yoon and Wise (2014) about the affective experience of color combination based on the color image scale is meaningful in that they used multiple combinations of color as visual stimuli applied to a three-dimensional physical environment. In a study of the impact of color on the perception of store luxury, emotions, and store preference, Cho and Lee (2017) found that color combinations perceived as high luxury (HL) impact pleasure, arousal, and store preference more than those perceived as low luxury (LL). The reason for the dearth of studies on color combination may be the result of the complexity involved in selecting appropriate colors and the difficulty in producing visual stimuli to test the impact of color on emotion by controlling other attributes.

In assessing the human response to environmental stimuli, pleasure and arousal have been the two significant emotional dimensions believed to influence behavior (Baker et al., 2002). In order to understand how pleasure and arousal change depending on spatial applications of a color combination, eye-tracking technology can be an effective tool to measure eye movements like fixations (stops) and saccades (jumps or moves). Eye-tracking technology provides researchers with objective data on the viewer’s subjective emotions and cognitive processes occurring in the brain, facilitating understanding of human gaze behavior. The eye-tracking technique allows a researcher to identify where and how long a person looks in the targeted area (dwell time) as well as the time-based sequence of eye movement from one location to another. Previous studies on gaze behavior and visual attention to stimuli have provided useful insights into understanding the relationship between viewers’ tendency to look at certain stimuli and their preferences. In eye-tracking research, longer fixation in a particular area of interest (AOI) is typically considered indicative of greater interest and engagement (e.g., Just and Carpenter, 1976; Poole et al., 2005). AOI can be created “*a priori* or *post hoc* as geometric or free-form shapes around products, items, or any other section of the image the researcher designers to analyze” (Huddleston et al., 2015, p. 568). *Dwell time*, also called visit duration, is defined as the “sum (all fixations and saccades within an AOI for all selected participants)/number of selected participants” (SensoMotoricInstruments, 2017, p. 243). Total visit duration, which is the same as dwell time, can be interpreted as a measure of “cognitive processing (thought) through attention” (Huddleston et al., 2015, p. 568).

Eye-tracking data can be used to interpret the existence of gaze bias in a given task. The gaze bias effect refers to the tendency to look longer at certain preferred stimuli, and an individual’s gaze behavior is known to be closely related to a viewer’s preference and decision making (Shimojo et al., 2003; Saito et al., 2017). Gaze bias is also involved in viewing three-dimensional spatial visual stimuli and spatial decision making (Wiener et al., 2012). Meanwhile, pupillometric data reflect the brain’s cognitive and emotional processes (Granholm and Steinhauer, 2004), especially the arousal dimension. The diameter of the pupillary aperture of the eye tends to increase in an arousal state and decrease in an unpleasant state (Hess and Polt, 1960). Some more recent studies reported that pupil size increases in emotionally arousing materials irrespective of hedonic liking (Bradley et al., 2017). Likewise, eye-tracking technology may provide useful information about subtle differences in emotional responses during a spatial search by demonstrating how eye movement and pupil size are influenced by spatial visual stimuli where color combinations are used. In addition, because two factors influence visual attention—the top–down factor (individual traits) and the bottom–up factor (physical characteristics of stimuli) (Wedel and Pieters, 2008)—the identification of participants with similar demographic characteristics is necessary.

In summary, available literature suggests the following:

First, previous researchers investigating the role of colors on emotion have mainly used a single color, not color combinations. Only a small number of them have investigated the impact of color combinations on emotion or preference; therefore, more studies using color combinations are necessary. Second, an understanding of the way one color combination may change emotional and cognitive response when applied in a space is needed. Because many interior spaces one encounters and uses consist of several colors, examining users’ responses to color combinations is worthwhile. Third, the impact of a single color combination applied to a physical environment on the perception of luxury has rarely been studied, and thus requires attention. With one color combination, whether the perception of luxury or intent to stay differs depending on where the color is applied, such as floor, wall, or furniture, is not well known. Fourth, using eye-tracking technology may reveal users’ tendencies in gaze behavior with regard to how spatial color combinations are applied. The association between gaze behavior and verbal response indicating the most luxurious space and PS may reveal the user’s behavior underlying the designation of a pleasurable experience. To our knowledge, this study is a first attempt to use eye-tracking technology to investigate the role that the spatial application of a color combination plays in affecting spatial color efficacy leading to the perception of luxury and the intent to stay.

Therefore, in this exploratory study, we hypothesized the following:

H1: Spatial color efficacy and spatial applications of a color combination: When applied differently in a three-dimensional space, a color combination will create different emotional and cognitive responses. This phenomenon will be observed in verbal response about the selection of PL and PS as well as the dwell time and the pupil size in eye-tracking data during free viewing and the selection tasks.

H2: Spatial color efficacy and PL: Some spatial applications of the same color combination will be more effective than others in affecting the viewer’s PL in a retail space. Dwell time in the selection task will correlate with the selection of PL, and the pupil size will correlate with PL.

H3: Spatial color efficacy and PS: Some spatial applications of the same color combination will be more effective than others in affecting the viewer’s PS in a retail space. Dwell time in the selection task will correlate with the selection of PS, and the pupil size will correlate with PS.

H4: Depending on how a color combination is applied in a space, even a LL color combination will create positive emotional and cognitive responses, influencing PL and PS.

**FIGURE 1:**
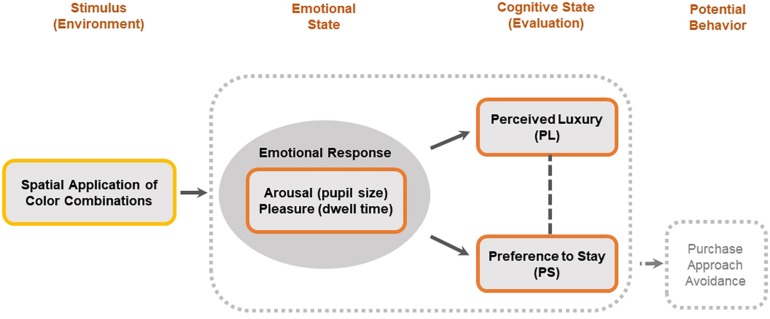
Conceptual framework of spatial color efficacy.

Figure 1 provides a summary of a conceptual framework of spatial color efficacy in this study. It shows that spatial color efficacy was investigated in two aspects: the effectiveness of spatial color on the perception of luxury and the effectiveness of spatial color on the intention to stay (preference). Spatial color applications consisting of two conditions of stimuli (HL color combination vs. LL color combination) were examined. Regarding emotional state, arousal was measured through the pupil size, and pleasure was measured through the dwell time. Regarding cognitive state, two dimensions of PL and PS were measured through the selection tasks. As a potential behavior, PS may be a critical indicator that leads to future purchase behavior. This framework is designed to clarify the understanding of human behavior in relation to spatial color efficacy based on the spatial color combination and potential decision-making process in a retail setting.

## Materials and Methods

### Experiment Material Development

In order to understand the spatial color efficacy on PL and PS, we first identified a HL color combination and a LL color combination. Then for each category, we created six different variations to examine how the participants’ emotional responses differ depending on where each color was applied. The color selection process was as follows.

#### Step 1: Selection of HL and LL Color Combinations

Color combinations conveying HL and LL were selected from previous research, in which one of the authors of the current paper had participated. Cho and Lee (2015) developed a hypothetical retail store and applied eight color combinations, having identified one HL and one LL color combination from their pilot study of a survey that asked 116 American and Korean college students to select HL and LL images. Cho and Lee (2017) confirmed in a survey of 218 adults that (a) the selected HL color combination was perceived as more luxurious than the selected LL color combination, and (b) the degree of perception of luxury increases pleasure and arousal, which ultimately increases store preference. In the current study, we used the same HL and LL color combinations that Cho and Lee (2017) used in their research as our initial base color combinations.

#### Step 2: Stimuli Development

The HL and LL combinations were applied to an image of a hypothetical retail store, developed with a 3D-rendering program, Sketch UP, and REVIT. The hypothetical store featured floor, wall shelves, mirror, ceiling, three display tables, a cashier’s desk, and two chairs. The size of the store was typical for a retail store, with 3.3 m in height, 18 m in depth, and 9.1 m in width. Appropriate ceiling and wall lighting for product display was applied to achieve a natural, typical look for a store. White tones were applied to ceiling. Three color combinations were applied as follows: Hue 1 to the floor (FL), Hue 2 to the main wall and furniture (WF), and Hue 3 to the recessed wall and seating (RS). In addition, to make the wall shelves visible, Hues 2 and 3 were applied to the shelves on the main wall and recessed wall to provide contrast. Thus, six variations of spatial applications were generated by applying Hues 1, 2, and 3 to the FL, WF, and RS in different ways. Table 1 provides a summary of a hypothetical store space and the visual stimuli (HL and LL combinations) used in the experiment.

**TABLE 1:** Basic structure of a hypothetical store and HL, LL visual stimuli.

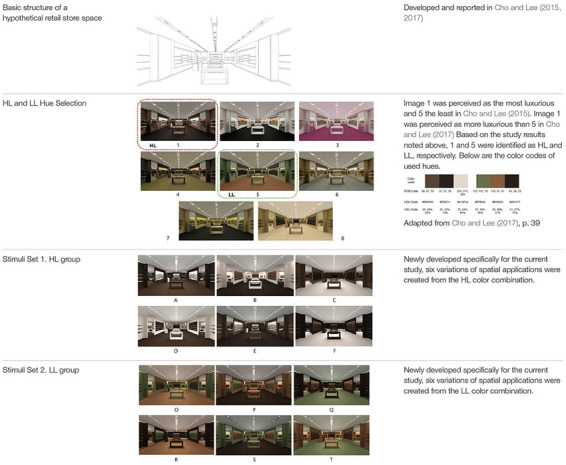	

### Participants

A total of 26 interior design majors at a university in South Korea voluntarily participated in the study. Interior design majors were selected because they are particularly qualified to participate in this study because they can be more sensitive to colors than laypersons as they have knowledge of color theory; in fact, the literature shows that designers and laypersons are known to differ in the way they look at buildings (Gifford et al., 2002). The research was announced to one interior design program in which one of the authors teaches, and students voluntarily signed up for the experiment. This study was conducted ethically with the approval of the Institutional Review Board (IRB).

### Experiment Setting and Procedure

The experiment was conducted in a quiet area in a small conference room with a desk and four chairs but no window in order to maintain same temperature, humidity, and light so that participants could focus on the experiment without disruption. During the experiment, participants sat in front of a 24-inch monitor (i.e., 537.6 mm × 296.5 mm without frame) equipped with an eye tracker placed approximately 65 cm from the monitor following the recommendations of the manufacturer. The luminance of the monitor screen was equalized in order to maintain identical brightness of the screen for the experiment. The eye-tracking device was the Remote Eye-Tracking Device Professional (RED) by SensoMotoric Instruments (SMI). The principal investigator and an assistant monitored and administered the experiment. The researchers sat next to the participant, looking at the laptop where the experiment program was installed. The eye-tracking data were stored with 60 Hz, which yields a total of 1,200 pieces of raw gaze data during 20 s per each stimulus. The experiment procedure was as follows. First, the calibration and validation process was conducted. In order to check the accuracy of data collection and precision, we maintained the deviation of tracking data below 0.5° on the X, Y axis. Second, an image was provided as a test for participants to become familiar with the setting. Then the experiment assistant explained the procedure to the participant.

The procedures of the main experiment were as follows:

(1) Participants looked at each image in the HL group displayed on a computer screen for 20 s per image.

(2) Then the six HL images were displayed together onscreen for free viewing (FV) with no task specified for 20 s.

(3) The six HL images were displayed together onscreen for 20 s, and participants were asked to evaluate the degree of luxuriousness of the images.

(4) The participants were asked to choose what they believed to be the most and least luxurious spaces and provide a verbal response about PL, explaining reasons for their selection with no time limit.

(5) The six HL images were displayed together onscreen again for 20 s, and participants were asked to evaluate the degree to which they preferred to remain in the space, or PS.

(6) The participants were asked to choose what they believed to be the space in which they would most and least prefer to stay and provide a verbal response about their PS, explaining reasons for their selection with no time limit.

The same procedure was repeated with the LL stimuli. Total duration for the experiment for each participant varied from 10 to 13 min because the verbal response times varied per individual.

**FIGURE 2:**
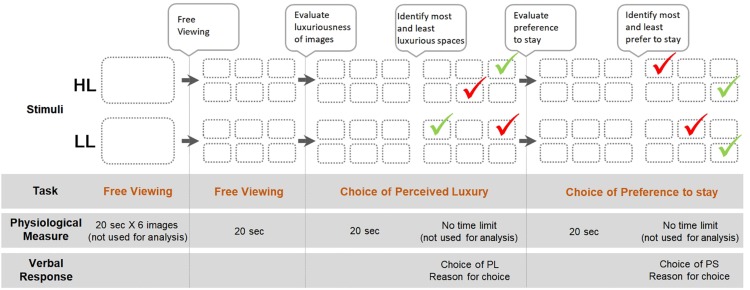
A diagram of experiment procedure.

At each step, the participants’ eye movements were recorded to determine whether gaze bias existed during the tasks and how the participants’ tendency to look at certain images related to their choices. Their eye activity data—dwell time and pupil size—were collected using the eye tracker and analyzed using SPSS; the frequency of verbal responses regarding the most luxurious image and the image of the space in which they most wanted to stay were counted. The measures of gaze behavior (dwell time) and arousal (pupil size) were examined during free viewing and during the two tasks in which they identified their PL and PS. Figure 2 shows the experiment procedure in a diagram format.

## Results

### Accuracy and Precision of Data Check

Checking the accuracy and precision of the obtained eye-tracking data is essential to maintain the quality of the data. *Accuracy* denotes “the difference between true and recorded gaze direction,” and *precision* denotes “how consistent calculated gaze points are, when the true gaze direction is constant” (Holmqvist et al., 2012). The two criteria examined in the eye-tracking data were the tracking ratio and the degree of eye deviation. The tracking ratio is the “[n]umber of non-zero gaze positions divided by sampling frequency multiplied by run duration, expressed in percent” (SensoMotoricInstruments, 2017, p. 347); that is, it shows how much gaze data were collected from the participants. The higher the tracking ratio, the more real gaze data are collected. The tracking ratio can reveal the accuracy and reliability of the data, and more than 80% is considered reliable (Shin and Shin, 2013). Precision can be explained with the degree of eye deviation. *Calibration X deviation* means deviation on X for eye data (SensoMotoricInstruments, 2017); thus, one participant can produce four deviation degrees: right eye X deviation, right eye Y deviation, left eye X deviation, and left eye Y deviation. The recommended degree is less than 0.5°. After removing data from two participants with less than 80% tracking ratio and the other four with more than 0.5° of eye deviations, a total of 20 participants’ data were selected and analyzed (15 females and 5 males). As a result, the proportion of dismissed participants was 23%, that is, 6 of the original 26.

### Spatial Color Efficacy Observed by Verbal Response

#### Perceived Luxury

First, participants’ verbally delivered responses about PL were analyzed in terms of the frequency of responses and the reason for selection. Table 2 includes a summary of the participants’ responses regarding the group of HL images, consisting of a three-hue combination: dark brown, brown, and ivory.

**TABLE 2:** Frequency of image chosen for PL and reason for selection in HL.

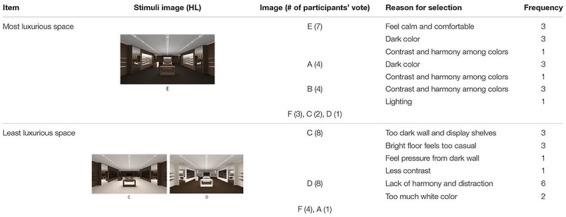	

Among the six images, E was chosen as the most luxurious space by seven participants. It featured a dark brown FL, brown WF, and ivory RS. Reasons for selecting E included dark color and a harmonized look with a nice contrast of colors; participants said they would feel calm and comfortable in this setting. In contrast, C and D were chosen as the least luxurious image by eight participants each. C featured an ivory FL, brown WF, and dark brown RS; D featured a brown FL, ivory WF, and dark brown RS. Participants stated that in C they would feel uncomfortable because of its excessively dark wall and display shelves lacked contrast, and the dark wall and bright floor created too much contrast; in D they said they would feel distracted and uncomfortable because of the lack of harmony among colors and the excessively bright color used on the wall. When comparing spatial color used in E (most), and C and D (least), E had a relatively larger amount of dark color used on both floor and main walls; C and D had a stronger contrast between the wall and the floor.

The LL color group featured brown, orange (in medium dark shade), and green (in medium dark shade). Table 3 offers a summary of images selected as most luxurious, the frequency of responses, and reasons for selection.

**TABLE 3:** Frequency of image chosen for PL and reason for selection in LL.

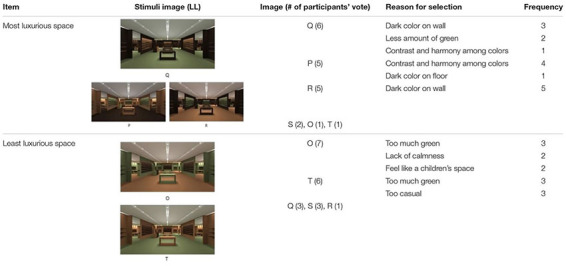	

*Note: Total amount of vote is more than the number of students as some students voted two images.*

Among the six images, Q was chosen as the most luxurious space by six participants. Q featured a green FL, brown WF, and orange RS. Reasons for selecting Q included its use of dark color on the walls and a small amount of green used in the space. P and R were also highly rated as the most luxurious space with five votes each. P featured a brown FL, orange WF, and green RS; R featured an orange FL, brown WF, and green RS. Reasons for selecting P and R included contrast and harmony among colors as well as the dark color used in the space, which created a luxurious look. In contrast, as the least luxurious image, O was selected by seven participants; it featured an orange FL, green WF, and brown RS. Participants stated that O lacked calmness because of the excessive amount of green used in the space. T was also frequently selected as least luxurious with six votes; it featured a green FL, orange WF, and brown RS. Respondents also mentioned excessive use of green as the reason for lack of luxuriousness; they stated that green seemed more appropriate for a children’s space and felt more casual than luxurious. When comparing the spatial color used in Q, P, and R (most luxurious), and O and T (least luxurious), Q, P, and R featured relatively larger amounts of dark color used, such as on the floor or the main wall; but in O and T, only the RS was a dark color.

Response results indicate that green applied to the wall in an interior space diminishes the perception of luxury, and brown walls facilitate the perception of luxury. Orange was not mentioned as a reason for luxuriousness.

In summary, participants’ responses regarding PL changed according to different ways of application of a color combination in a physical environment. Throughout the two sets of experiment results, the common features of PL emerged as follows. When same color combinations are applied, people tended to feel (a) high PL when a relatively large amount of the space was of a brown with darker tone; (b) high PL when a brown hue with darker tone was applied on the floor than on the wall or other interior elements; (c) low PL when the floor is lighter than the walls or other interior elements; (d) high PL when a lighter color appears on a recessed wall and a darker color appears on the main wall than vice versa; and (e) low PL resulting from too much contrast between colors or lack of contrast.

#### Preference to Stay

Participants’ verbally delivered choices of PS were analyzed in terms of the frequency of responses and reasons for selection. Table 4 is a summary of the frequency of images chosen for PS in the HL group.

**TABLE 4:** Frequency of image chosen for PS and reason for selection in HL.

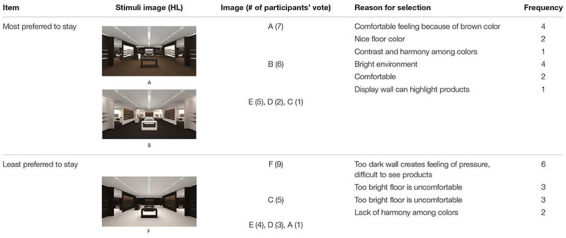	

Among the six images, A was chosen by seven participants as the space in which they would most prefer to stay. A featured a brown FL, dark brown WF, and ivory RS. Reasons for selecting A included nice brown colors, which felt calm and comfortable. B was also highly regarded by six participants as the space in which they would most prefer to stay. It featured a dark brown FL, ivory WF, and brown RS. Participants responded that a bright color yields a comfortable environment; a bright wall and dark floor seem to highlight products displayed well. In contrast, a space in which participants least preferred to stay was image F, featuring an ivory FL, dark brown WF, and brown RS. Participants reported that F featured too dark walls and light floor with too much contrast but a lack of contrast on the main and recessed walls, making them feel pressure from the dark walls and uncomfortable from the light floor to use as a space.

Table 5 presents a summary of the frequency of images chosen for PS in the LL group.

**TABLE 5:** Frequency of image chosen for PS and reason for selection in LL.

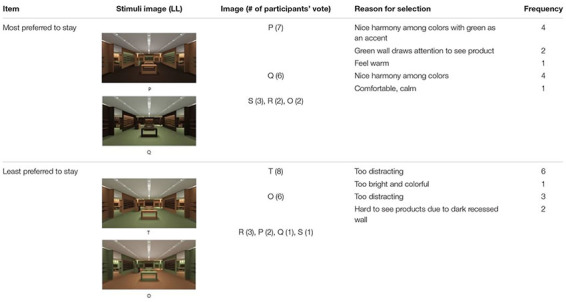	

Among the six images, P was chosen as the space where participants would most prefer to stay with seven votes. P featured a brown FL, orange WF, and green RS. Reasons for selecting P included its nice harmony among colors with green as an accent, making them feel comfortable to stay. Q was also highly ranked as a place they preferred to stay, chosen six times and featuring a green FL, brown WF, and orange RS. Participants stated that the brown walls made them feel comfortable staying. As the space in which they least preferred to stay, T garnered eight votes and featured a green FL, orange on WF, and brown RS. Participants reported that the bright orange wall and green floors were too distracting and colorful, making them feel uncomfortable about staying. The other space identified as the one in which they least preferred to stay was O, featuring an orange FL, green WF, and brown RS. The commonalities between T and O included brown applied only in small amounts on the RS, and green and orange as the main colors in the space. Participants stated that T and O seemed like children’s spaces, such as playgrounds and elementary schools, not spaces for adults.

In summary, one color combination applied in different ways in a physical environment can change the participants’ responses regarding PS. Throughout the two sets of experiment results, the common features of PS emerged as follows. When same color combinations are applied, (a) people tend to prefer a proper level of contrast, not too much contrast, or lack of contrast between colors; and (b) the use of similar tones of two distinctive colors in the majority of the space is unlikely to be preferred.

### Spatial Color Efficacy Captured by Eye-Tracking While Viewing Collection of Six Images

#### Free Viewing and Gaze Behavior

In order to understand varying degrees of spatial color efficacy in variations of spatial application of the same color combination, participants’ emotional responses were examined using dwell time and pupil size.

**FIGURE 3:**
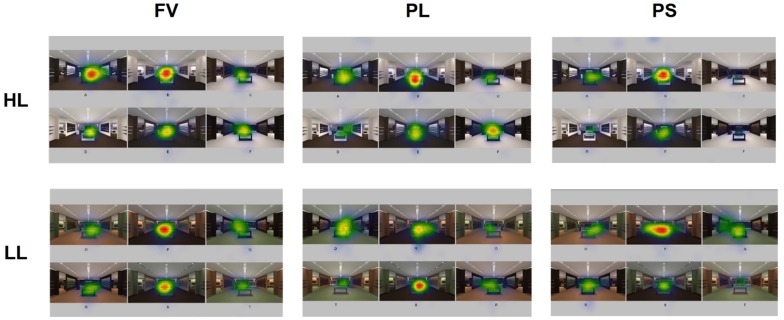
Heat maps of experiment stimuli.

**FIGURE 4:**
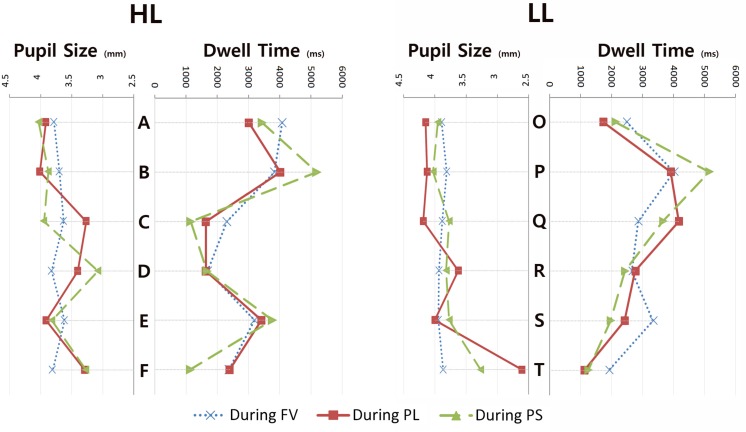
Dwell time and pupil size in HL and LL settings.

When analyzing gaze behavior in viewing the collection of six images together, we set up each image as an AOI and examined the dwell time and the pupil size. Following recommendations from the SMI manual, we used a dwell time longer than 0.08 s (default value) for analysis. We calculated the dwell time for each image during (a) free viewing (FV) of the stimuli without a specific task given; (b) search for a space with PL; and (c) search for a space where participants preferred to stay (PS). Figure 3 presents screen captures of heat maps of eye-tracking data. Figure 4 presents a summary of the dwell time and the pupil size when viewing spaces considered HL and LL.

The heat maps show “gaze patterns over the stimulus image visualized as a colored map” (SensoMotoricInstruments, 2017, p. 101). They show data distribution and different intensity of the visual observation (Špakov and Miniotas, 2007). They are generated based on fixation hits: blue indicates less hits, while red means most hits in the three-color coding system. Figure 3 shows that, during free viewing of HL, participants’ visual attentions were more on images A and B than other images, and these two were chosen as the spaces where participants would most prefer to stay. In addition, during free viewing of LL, participants’ visual attentions were more on images P, the space where participants would most prefer to stay, than other images.

Figure 4 shows that the dwell time and the pupil size when considering PL as well as PS tended to be clearly different across six images. To illustrate, in HL compared to the other two occasions (FV and the search for PL), in the search for PS, the dwell time for B was relatively long, and dwell times for C and F were relatively short. In addition, among LL images, compared to the other two occasions (FV and the search for PL), in the search for PS, the dwell time for P was relatively long, and dwell times for S and T were relatively short, indicating that more gaze bias exists in preference decisions than in decisions of perception of luxury or free viewing. Figure 4 also shows that the distribution of the pupil size resembles that of the dwell time for determining PL. In contrast, the distribution of the pupil size does not resemble that of the dwell time during FV.

In terms of HL, the image with the longest dwell time during FV was A, followed by B and E. In fact, participants also cited A as the space where they would most prefer to stay, followed by B and E, in the same order. When determining PL, the image with the longest dwell time was B, followed by E and A. B was actually the image listed as the second most luxurious space. When determining PS, the image with the longest dwell time was B followed by E and A. In fact, B was the image listed as the second most preferred space with just a one vote difference from A. Participants spent the longest time on B when searching for the most luxurious space as well as the most preferred space, but the selection of E as the most luxurious space and the selection of B as the space where they would most prefer to stay may indicate that people debated their decision among A, B, and E. In contrast, dwell times for C and D, which were selected as the least luxurious space, were the shortest during the choice of PL. Likewise, F, selected as the space where participants would least prefer to stay, had the shortest dwell time during the choice of PS. The results indicate that some of the images are more visually engaging to the viewers than others.

In the viewing of the LL group, the image with the longest dwell time during FV was P, which was actually selected as the space where most would prefer to stay. During the search to determine PL, the image with the longest dwell time was Q, which was selected as the image most luxurious. During the search to determine PS, the image with the longest dwell time was P, which was actually selected as the space where most prefer to stay. Compared to the other two occasions, in the search for PS, the dwell time for P was notably long, indicating that depending on the spatial application, a space developed based on a low luxurious color combination can be visually appealing and engaging.

In addition, in order to examine any statistically significant relationships among dwell times and choices for PL and PS, a correlation analysis was conducted. The results are reported in Table 6.

**TABLE 6:** Correlation analysis between dwell time and response for HL and LL.

**HL**		**Dwell time during PL**						**Dwell time during PS**					
		**A**	**B**	**C**	**D**	**E**	**F**	**A**	**B**	**C**	**D**	**E**	**F**
Choice of PL	A	0.946**											
	B		0.879**										
	C			0.852**									
	D				0.451*								
	E					0.678**							
	F						0.266						
Choice of PS	A							0.625**					
	B								0.803**				
	C									0.590**			
	D										0.718**		
	E											0.790**	
	F												−

**LL**		**Dwell time during PL**						**Dwell time during PS**					
		**O**	**P**	**Q**	**R**	**S**	**T**	**O**	**P**	**Q**	**R**	**S**	**T**

Choice of PL	O	0.927**											
	P		0.906**										
	Q			0.879**									
	R				0.775**								
	S					0.933**							
	T						0.919**						
Choice of PS	O							0.794**					
	P								0.819**				
	Q									0.860**			
	R										0.787**		
	S											0.796**	
	T												−

*Correlation is significant at the 0.05 level. **Correlation is significant at the 0.01 level (two-tailed).

The results show that positive correlations were found between the dwell time and the choice of PL in images A, B, C, D, and E. In addition, positive correlations were found between the dwell time and the choice of PS in images A, B, C, D, and E as well as in the dwell time in free viewing and their choice of PL in images A, D, E, and F. No correlation was found, however, between the dwell time in FV and the choice of PS.

Among LL stimuli, the results of the correlation analysis show that positive correlations were found between the dwell time and the choice of PL in all six images. In addition, positive correlations were found between the dwell time and the choice of PS in images O, P, Q, R, and S and between the dwell time in free viewing and their choice of PL in images P, R, and S.

#### Pupil Size Differences

In order to determine whether the participant’s arousal state changes during viewing different spatial applications of the same color combination, we also examined pupil size changes. According to the literature, the pupil size can be an indicator of emotional response, arousal, and strong interest (Sirois and Brisson, 2014; Bell et al., 2018; Mathôt, 2018); therefore, the pupil size for each AOI was calculated during FL, PL, and PS.

In order to understand whether the pupil size differs across images, a paired sample t-test was conducted. The results showed that during the choice of PL, the pupil size while viewing B (*M* = 4.01, *SD* = 0.52) and C (*M* = 3.27, *SD* = 1.73) was different but at a level only marginally statistically not significant [*t*(19) = 2.07, *p* = 0.052].

During the choice of PS, a significant difference in the pupil size was found between A (*M* = 4.03, *SD* = 0.55) and D (*M* = 3.08, *SD* = 1.62); *t*(19) = 2.15, *p* = 0.04; and between A (*M* = 4.23, *SD* = 0.70) and *F*(*M* = 3.92, *SD* = 1.04) at a level marginally not statistically significant [*t*(19) = 2.00, *p* = 0.059]. During the choice of PS, A was actually the image of the space where participants most preferred to stay, and F the least, which means the pupil size differed when looking at images of spaces where participants most and least preferred to stay. The pupil size was larger when participants looked at the images of spaces where they most preferred to stay than least.

Among LL stimuli, during the choice of PL, a significant difference in the pupil size was found between O (*M* = 4.15, *SD* = 0.48) and T (*M* = 2.61, *SD* = 2.01); *t*(19) = 3.26, *p* = 0.004; between P (*M* = 4.12, *SD* = 0.60) and T; *t*(19) = 3.42, *p* = 0.003; between Q (*M* = 4.19, *SD* = 0.51) and T; *t*(19) = 3.33, *p* = 0.003; and between S (*M* = 4.00, *SD* = 1.09) and T; *t*(19) = 2.63, *p* = 0.016. The pupil size was larger when participants looked at the images of most luxurious than least. During the choice of PS, the pupil size differed between O (*M* = 3.95, *SD* = 1.05) and T (*M* = 3.27, *SD* = 1.74) and between P (*M* = 3.82, *SD* = 1.41) and T but at a level only marginally not statistically significant [*t*(19) = 2.02, *p* = 0.059, and *t*(19) = 2.02, *p* = 0.057 each]. In fact, T was selected as the image of the space that participants least preferred to stay as well the second least luxurious. Q was most luxurious, and P was the image of the space where participants most preferred to stay. Although not at a statistically significant level, data showed that the pupil size differed when looking at images of spaces where participants most and least preferred to stay.

In summary, how research results supported hypotheses appears below:

H1: Spatial color efficacy and spatial applications of a color combination: When applied differently in a three-dimensional space, the same color combination will create different emotional and cognitive responses. This phenomenon will be observed in verbal response about the selection of PL and PS as well as the dwell time and the pupil size in eye-tracking data during free viewing and the selection tasks. → Supported

H2: Spatial color efficacy and PL: Some spatial application of the same color combination will be more effective than others in affecting the viewer’s PL in a retail space. The dwell time in the selection task will correlate with the selection of PL, and the pupil size will correlate with PL. → Partially supported. The dwell time and the selection correlated, but the pupil size did not correlate with PL.

H3: Spatial color efficacy and PS: Some spatial application of the same color combination will be more effective than others in affecting the viewer’s PS in a retail space. The dwell time in the selection task will correlate with the selection of PS, and the pupil size will correlate with PS. → Partially supported. The dwell time and the selection correlated, but the pupil size did not correlate with PS.

H4: Depending on how a color combination is applied in a space, even a LL color combination will create positive emotional and cognitive responses, influencing PL and PS. → Supported

## Discussion

### Spatial Color Efficacy on Perceived Luxury and Preference to Stay

Our findings in this research demonstrate that spatial color efficacy in various applications of one color combination could vary; furthermore, this research revealed that spatial color efficacy resulting from spatial application of one color combination may vary in affecting PL as well as PS. We also demonstrate that, depending on spatial application, even a LL color combination can enhance positive emotional response leading to PL and PS. These results address the limitation of relying only on color combination swatches in spatial design and suggest vigilant attention to detailed aspects of spatial color efficacy in designing retail interior spaces. In fact, subtle changes in the spatial application of a color combination can create compelling pleasurable experience and may influence the customers’ perception and behavior. Therefore, when designing a retail interior space, generating variations of spatial application of initial color combinations as a trial run and assessing the detailed atmospheric quality created by each spatial application will be a meaningful exercise. A palette of three colors may appear as a unitary color combination, but innumerable possibilities of potential spatial variations are embedded, which could lead to diverse emotional and cognitive responses. To our knowledge, this is the first study of the relationship between PL and PS and the application of variations of one color combination in a retail environment, in particular, using eye-tracking technology.

When a color combination was applied differently on the floor, main or recessed wall, and furniture, certain tendencies were observed; and thus, we provide the following recommendations for designers and marketers to achieve high levels of PL and PS:

• When a relatively large amount of the space is filled with a darker hue, particularly on the floor or main walls, the perceived level of luxury tends to be high. A large amount of space in a dark color is likely to enhance the perception of luxury.

• Excessive use of green and orange tones can cause distraction, but using these two in reduced amounts and brown in greater amounts of space generally enhances PL. With a dark display background, a green hue-based combination can enhance the feeling of luxury.

• Creating a bright green wall can diminish PL: Projection of green on a large wall may decrease PL.

• Extreme contrast among components, such as walls and floors, or lack of contrast generally decreases pleasurable response and desire to stay. It seems that PS and the amount of contrast seem to have an inverted U-shape relationship. Achieving an appropriate level of contrast is a key to enhance the pleasurable experience.

• Wood (brown) tones make the atmosphere comfortable and increases the desire to stay.

• The space perceived most luxurious and the space where people most want to stay did not always correlate.

Then what are the possible reasons behind such tendencies? The participants’ preference for darker color on surrounding walls and floor may be explained by Stenglin’s (2008) binding theory, according to which feeling too unbound by a lack of vertical enclosure causes insecurity, and an extreme level of binding created by spatial enclosure can evoke claustrophobic responses. In contrast, an appropriate level of spatial enclosure can “produce comfort zones of security or freedom” (p. 425). A sense of enclosure and a sense of privacy created by spatial enclosure (Ching, 1996) are important affective dimensions that cause the feeling of comfort and security in a space. Although binding theory does not address the relationship between the affect created by applications of color and spatial enclosure, depending on the property of color, spatial planes could appear approaching or receding and thereby affect the visual emotional perception of the enclosure of a space. One result of this study regarding viewers’ preference for dark brown on the floor aligns with Rasmussen’s (1964) explanation of “impression of gravity” (p. 219) that can be achieved by using a brown hue on a floor.

Participants’ preference for similar hues in varying tones (e.g., brown and dark brown in image A) in a majority of spaces can be explained by the similarity-based model of color relationship suggested by Deng et al. (2010). In an experiment in which participants were asked to choose up to seven colors for seven components of a shoe design, Deng et al. (2010) found that people used relatively small numbers of color palettes. They also found that people like to achieve overall visual congruence by using colors relatively close or exactly matching in the CIELAB color space, and with congruency as a background, people use different hues as a salient figure. Deng et al. (2010) argued that the use of similar colors can increase figural goodness, coherence, unity, and easy perception with less cognitive load, a tendency more strongly supporting the influence of a visual coherence perspective than an optimal arousal perspective on aesthetic preference. When interpreting the similarity-based model of color relationship in the three-dimensional interior space, we understand that people may like background colors of similar hues with varying tones (lightness) and one distinctive accent color. In the current research, participants did not prefer spaces of distinctive hues with similar tones (e.g., orange and green) used in the majority of spaces as observed in image O or T, aligning with the results of the study by Deng et al. (2010) on the infrequent use of colors of the same degree of lightness with hues or saturations of considerable difference. In addition, the finding of the preference of participants in the current study for a proper level of contrast instead of extreme contrast or lack of contrast between the wall and the floor can be explained from the perspective of the congruence model because too much contrast makes the overall space seem disunified and complex.

### Gaze Bias

This research supports the gaze bias theory, addressed by many previous researchers. The results of this study demonstrate that viewers’ gaze behavior is related to their preference or choice. The tendency toward viewing certain images longer than others existed in the selection tasks. The dwell time in the task of searching to determine PL correlated with the choice of luxurious space; and the dwell time in the selection task of indicating PS also correlated with the choice of space in which participants preferred to stay. This finding aligns with those in previous studies on gaze bias and preference (Shimojo et al., 2003; Saito et al., 2017). Even though the pupil size did not correlate to the point of statistical significance with the dwell time or the selection, the tendency showed that patterns in the dwell time and the pupil size have some similarity in distribution. This result suggests that depending on the application of the colors, a single color combination can create different emotional and cognitive responses; therefore, some of the applications can appear more attractive than others and visually engaging for the viewers.

### Implications, Limitations, and Future Research

This study provides several implications to designers, educators, and researchers. First, findings from this research suggest a useful guideline for designers in planning retail stores to achieve desirable levels of luxury. Second, this research provides a recommendation to design educators who teach color theory and environmental psychology to incorporate both color combinations and spatial application. Color use in a space differs from that on a product or an object because people are positioned inside three-dimensional spatial elements. In understanding the human emotional and cognitive response to color in a space, consideration of multidimensional phenomena created by the application of colors in a physical space is crucial. In a three-dimensional space, the overall proportion and composition of multiple components—walls, floors, and objects—are perceived along with colors. Some elements are under feet; others, overhead; and still others, in the front or on the side. The surfaces of these colored components in relation to the body can alter visual perceptions. Sometimes students learn about color theory only on small color swatches, and they do not delve into the possibility of diverse applications of one color combination in three-dimensional space and the emotional, cognitive, and behavioral responses generated from such application. Developing software that could generate multiple variations of spatial color applications for students to view or a software program using augmented reality (AR) to apply color combinations to real space and see the effect easily would be of considerable help in nurturing the perceptual understanding and knowledge of color application for the pleasurable experience of space. Third, this research provides insight to researchers on the use of eye-tracking technology on the study of impact of color on emotion and behavior. As one of the physiological measurements, eye-tracking technology can be a strong link to reveal behaviors underlying the decision-making process.

Three limitations are present in this study. First, the number of participants was small and limited to interior design students in one country. Students may not have the economic and financial capacity to afford luxury goods, so some gap may exist between actual luxury consumers and members of the participant group. However, as millennials, born between 1980 and 2000, occupy a substantial market in Korea (Ahn, 2019) and overseas (Lee, 2019) and the concept of luxury expands to highlight self-concept and experiential quality instead of exclusiveness or inaccessibility (Chandon et al., 2016; de Kerviler and Rodriguez, 2019), a study with college students as participants is still meaningful. The reason for engaging interior design major students as participants was to study individuals with similar characteristics who would reveal the gaze behavior of a particular group. Based on the findings of this study, future studies could expand to participant groups with diverse demographic backgrounds to achieve generalization. Second, in the current study, only two color combinations were used for the study of spatial color efficacy. Incorporating more diverse color combinations as stimuli in experiments will be helpful in observing gaze behavior and identifying commonalities of the space perceived as luxurious and the one in which people preferred to stay; this information can be developed into a spatial color efficacy theory in interior design. Third, we did not include any questions on purchase intention in this study because no specific product was designated.

Future research that includes purchase intention will be helpful to reveal the link between verbal and physiological response and purchase behavior. In addition, as spatial color efficacy was revealed, in order to more systematically investigate attributes that contribute to PL and PS, a study using choice modeling theory will be meaningful. Choice modeling theory will allow researchers to examine how and what attributes among those found in this research—such as contrast among colors, darkness in tones, and similarity-based color of relationship—influence participants’ decision making for PL and PS. Moreover, examining participants’ own meaning of luxury was beyond the scope of this research. Linking participants’ perception of luxury and verbal responses could reveal attributes contributing to PL and PS. Because both top-down factors (individual characteristics) as well as bottom-up factors (physical attributes of stimuli) are important in visual attention and environmental psychology, future researchers should systematically incorporate both factors.

## Data Availability Statement

The datasets generated for this study are available on request to the corresponding author.

## Ethics Statement

The studies involving human participants were reviewed and approved by Kyung Hee University. The patients/participants provided their written informed consent to participate in this study.

## Author Contributions

JC developed the study concept, collected the data, conducted the data analysis, drafted and reviewed the manuscript, and supervised the research. JS developed the conceptual framework, interpreted the data, drafted the manuscript, and extensively reviewed and edited the whole manuscript. All authors contributed to manuscript revision and read and approved the submitted version.

## Conflict of Interest

The authors declare that the research was conducted in the absence of any commercial or financial relationships that could be construed as a potential conflict of interest.
